# *Segniliparus* *rugosus* Infection, Australia

**DOI:** 10.3201/eid1504.081479

**Published:** 2009-04

**Authors:** Tarrant Hansen, Janelle Van Kerckhof, Peter Jelfs, Claire Wainwright, Pat Ryan, Chris Coulter

**Affiliations:** Mycobacterium Reference Laboratory, Brisbane, Queensland, Australia (T. Hansen, J. Van Kerckhof, C. Coulter); Westmead Hospital, Westmead, New South Wales, Australia (P. Jelfs); Royal Children's Hospital, Herston, Queensland, Australia (C. Wainwright); University of Queensland, Brisbane (C. Wainwright); Townsville Hospital, Townsville, Queensland, Australia (P. Ryan)

**Keywords:** Segniliparus rugosus, cystic fibrosis, acid-fast bacilli, letter

**To the Editor:** Recently, a female teenager with cystic fibrosis who resided in tropical north Queensland, Australia, was found to be infected with *Segniliparus rugosus*. She was homozygous for the deltaF508 mutation, had well-preserved lung function, and regularly played competitive sports. Unlike many cystic fibrosis patients, she did not have a history of chronic *Pseudomonas aeruginosa* infections, but *Stenotrophomonas maltophilia* and *Achromobacter xylosoxidans* had been previously isolated from her sputum. In May 2007, she described reduced exercise tolerance and increased cough with excess sputum production. Lung function testing showed modest spirometric decline. A computed tomographic scan of the chest showed significant mucus plugging and bronchiectasis, uncommon without previous *P. aeruginosa* infection. Sputum was 3+ smear positive for acid-fast bacilli (AFB), and *S. rugosus* was isolated from liquid culture. Empiric antimicrobial drug therapy was changed to rifabutin and co-trimoxazole because these drugs have been effective in previous cases (*1*). Clinically, the patient showed response to the treatment. After 12 months of treatment, her sputum was still 3+ positive for AFB, and *S. rugosus* was again found in culture. She was referred to a pediatric teaching hospital in Brisbane with worsening respiratory symptoms precipitated by influenza B infection. Antimicrobial drug therapy with intravenous imipenem, oral moxifloxacin, and co-trimoxazole for 2 weeks resulted in clinical improvement but little reduction in smear positivity.

The initial AFB smear-positive sputum specimen underwent routine decontamination with sodium hydroxide and neutralization and was inoculated into radiometric 12B vials (Becton Dickinson, Sparks, MD, USA). The culture was positive after 6 days’ incubation and was referred to the state reference laboratory in Brisbane. The culture was smeared and stained with the Ziehl-Neelsen method, which showed short, pale AFB.

A crude boil DNA preparation was made from the positive culture, and an in-house multiplex PCR to identify *Mycobacterium* spp. was performed (*2*). No PCR products were detected, and the *Mycobacterium* genus-specific band was absent. Similarly, no *Mycobacterium* genus band was detected by using a PCR-hybridization method (Common Mycobacteria line probe assay, Hain Lifesciences GmbH, Nehren, Germany) (*3*). DNA sequencing was performed on the *Segniliparus rugosus* strain NQ1 (GenBank accession no. FJ593188), and a 1,250-bp fragment of the ribosomal 16S loci was obtained. A BLAST search (GenBank) was then performed on the sequence information obtained. The DNA sequence gave a 100% match to that of *Segniliparus rugosus* (GenBank accession no. AY608920) (*4*).

Drug susceptibility testing (DST) was attempted by using disk diffusion with Mueller-Hinton plates but was unsuccessful because of insufficient growth and indistinct margins. A commercially available microbroth dilution assay was used to determine the MICs for 12 antimicrobial drugs. The Sensititre Broth MIC Method for Rapidly Growing Mycobacteria, Nocardia, and Other Aerobic Actinomycetes (Trek Diagnostic Systems, Cleveland, OH, USA) was used according to manufacturer’s instructions (*5*). However, problems still occurred because of inadequate growth of the organism using the testing media approved for mycobacteria (*6*,*7*). Superior results were obtained when the cation-adjusted Mueller-Hinton broth with TES buffer (Trek Diagnostic Systems), which is used routinely in this assay, was substituted with an internally prepared Middlebrook 7H9 broth (Becton Dickinson) (*1*). DST results are similar to those previously reported for other strains of *S. rugosus* (Table) (*1*).

The strain had rough, wrinkled colonial morphology on both blood agar and 7H11 plates. Growth was optimal at 35°C on 7H11 media. Arylsulfatase activity was weakly positive at 3 days and positive at 14 days. Growth on Lowenstein-Jensen (LJ) medium and on LJ with 5% sodium chloride medium occurred at 6–7 days. The strain was negative for both nitrate reductase and tween hydrolysis. Mycolic acid high-pressure liquid chromotography was performed (*8*), and the pattern obtained matched that of type strain CDC 945 (AY608920) previously reported (*4*). The pattern had a double cluster of adjoining eluting peaks with the last peak co-eluting with the internal high standard.

Only 3 other cases of *Segniliparus* spp. infection (none from Australia) have been reported. More remains to be learned about the effects of lung infections with *Segniliparus* spp. in cystic fibrosis patients. Although this patient improved clinically after treatment with antimicrobial drugs, she is still infected and will likely remain chronically infected. Because this genus is acid fast by the Ziehl-Neelsen method, laboratory workers and clinicians must be aware that AFB seen in pulmonary specimens from cystic fibrosis patients are not necessarily from the genus *Mycobacterium* and may be from the genus *Segniliparus*.

**Table:** Antimicrobial susceptibility patterns for *Segniliparus rugosus* strain in a cystic fibrosis patient, Australia

Antimicrobial agent	MIC, µg/mL
Amikacin	>128
Amoxicillin/clavulanic acid (ratio 2:1)	64/32
Cefoxitin	64
Ceftriaxone	>64
Ciprofloxacin	4
Clarithromycin	16
Gatifloxacin	0.5
Imipenem	1
Linezolid	64
Minocycline	>32
Tobramycin	>64
Trimethoprim/sulfamethoxazole	2/38
